# *Panax ginseng* Fruit Has Anti-Inflammatory Effect and Induces Osteogenic Differentiation by Regulating Nrf2/HO-1 Signaling Pathway in In Vitro and In Vivo Models of Periodontitis

**DOI:** 10.3390/antiox9121221

**Published:** 2020-12-03

**Authors:** Eun-Nam Kim, Tae-Young Kim, Eui Kyun Park, Jae-Young Kim, Gil-Saeng Jeong

**Affiliations:** 1 College of Pharmacy, Keimyung University, 1095 Dalgubeol-daero, Daegu 42601, Korea; enkimpharm@gmail.com; 2 Department of Biochemistry, School of Dentistry, IHBR, Kyungpook National University, 2177, Dalgubeol-daero, Jung-gu, Daegu 41940, Korea; tae09290@gmail.com (T.-Y.K.); jykim91@knu.ac.kr (J.-Y.K.); 3 Departments of Oral Pathology and Regenerative Medicine, School of Dentistry, Kyungpook National University, Daegu 41940, Korea; epark@knu.ac.kr

**Keywords:** periodontitis, *Porphyromonas gingivalis*, heme oxygenase-1, *Panax ginseng* fruit extract, osteogenic differentiation

## Abstract

Periodontitis is an infectious inflammatory disease of tissues around teeth that destroys connective tissues and is characterized by the loss of periodontal ligaments and alveolar bone. A new treatment strategy is needed owing to the limitations of the current surgical treatment method and the side effects of anti-inflammatory drugs. Therefore, here, we assessed whether *Panax ginseng* fruit extract (PGFE) is a new therapeutic agent for periodontitis in vitro and in vivo. According to the results, PGFE suppressed pro-inflammatory cytokines such as tumor necrosis factor-α, interleukin (IL)-1β, and IL-6, and pro-inflammatory mediators such as inducible nitric oxide synthase and cyclooxygenase-2 through heme oxygenase-1 expression in human periodontal ligament cells stimulated with *Porphyromonas gingivalis* lipopolysaccharide (PG-LPS). In addition, the osteogenic induction of human periodontal ligament cells was inhibited by PG-LPS, and protein and mRNA levels of osteogenic markers such as alkaline phosphatase, collagen type 1 (COL1), osteopontin (OPN), and runt-related transcription factor 2 (RUNX2) were increased. The efficacy of PGFE for inhibiting periodontitis in vitro was demonstrated in a representative in vitro model of periodontitis induced by ligature and PG-LPS. Subsequently, hematoxylin and eosin staining and micro-computed tomography of the euthanized experimental animal model confirmed suppressed periodontal inflammation, which is an important strategy for treating periodontitis and for recovering the resulting alveolar bone loss. Therefore, PGFE is a potential, novel therapeutic agent for periodontal diseases.

## 1. Introduction

Periodontitis results from chronic inflammatory reaction to bacterial plaques and is an epidemic that causes periodontal inflammation, which is characterized by the accumulation of tartar on the teeth and loss of the periodontal ligament (PDL) and alveolar bone [1,2], Periodontitis results in the upregulation of pro-inflammatory cytokines that stimulate inflammatory cells and destroy connective tissues, such as the PDL and alveolar bone [3]. In various pathogen-related molecular patterns, including inflammatory responses to lipopolysaccharides (LPS), tumor necrosis factor-α (TNF-α), interleukin (IL)-6, and IL-1 are activated by the formation of nitric oxide (NO) and prostaglandin E2 (PGE2), which are synthesized by inducible nitric oxide synthase (iNOS) and cyclooxygenase-2 (COX-2), respectively [4]. Previous studies have shown that LPS synthesized by certain subgingival Gram-negative organisms causes inflammation of the periodontal tissue, thus inducing the production of pro-inflammatory mediators, such as reactive oxygen species (ROS), cytokines, and prostaglandins [5,6]. In addition, one of the most important key factors responsible for periodontal destruction is the upregulation of inflammatory cytokines, including IL-1β, IL-6, and TNF-α, in the inflammatory sites of periodontal tissue; these cytokines are mainly involved in the pathogenesis of periodontitis [7]. Therefore, the most important treatment strategy for periodontitis is to suppress the production of these pro-inflammatory mediators and the expression of cytokines.

The PDL is a soft connective tissue located between the tooth cementum and alveolar bone. Similar to osteoblasts, it produces bone matrix proteins such as alkaline phosphatase (ALP), osteonectin, osteocalcin, and osteopontin (OPN). This special connective tissue supports and holds teeth in place [8,9]. Considering that the loss of alveolar bone due to chronic periodontitis eventually causes tooth loss, suppression of bone matrix protein loss in the PDL is another important target for the treatment of periodontitis [10,11].

The rate-limiting enzyme involved in the catabolism of heme is heme oxygenase (HO), producing equimolar amounts of free iron, carbon monoxide, and biliverdin. Of the three mammalian HO isoforms, HO-1 is a stress-responsive protein activated by various regulatory and protective mechanisms in cells. It is also a major oxidative stress marker induced by the activation of nuclear factor-erythroid 2-related factor 2 (Nrf2) [12,13]. Thus, the modulation of HO-1 activity can be a therapeutic strategy for several inflammatory conditions. According to previous studies, the accumulation of ROS further contributes to osteoporosis progression by inhibiting the activation of the Nrf2/HO-1 signaling pathway; moreover, as HO-1 expression is upregulated in the bone, it may be an important antioxidant defense mechanism [14,15]. However, only few studies have investigated the effect of HO-1 in inducing the differentiation of PDL cells into osteoblasts.

*Panax ginseng* C.A. Meyer, which belongs to the Araliaceae family, has long been known and widely used as a medicinal plant in Korea, Japan, and China [16]. It is one of the most widely used herbal medicines for the treatment of cardiovascular diseases, such as obstructive vasculitis, coronary artery disease, atherosclerosis, and cerebral infarction in China, Korea, and Russia [17]. The primary ingredients of *P. ginseng* include the protopanaxadiol ginsenoside Rb1 and the protopanaxatriol ginsenoside Re. These major ginsenosides exhibit anti-inflammatory, antioxidant, immune-modulating, and neuroprotective effects [18]. However, despite many studies on *P. ginseng* components and physiological activity, there are not many studies evaluating the pharmacological activity of *P. ginseng* fruit. Therefore, this study investigated the effect of *P. ginseng* fruit on periodontitis and alveolar bone loss in periodontitis-induced human PDL cells in vitro and in animal models in vivo.

## 2. Materials and Methods

### 2.1. Chemicals and Reagents

Minimum Essential Medium-Alpha (α-MEM), fetal bovine serum (FBS), and trypsin-ethylene diamine tetra acetic acid (EDTA) culture reagents were purchased from Gibco (Grand Island, NY, USA). 3-(4,5-Dimethylthiazol-2-yl)-2,5-diphenyltetrazoliumbromide (MTT) was obtained from Amresco Inc (Cleveland, OH, USA). TNF-α, IL-6, and ELISA (enzyme-linked immunosorbent assay) kits were purchased from R & D system (Minneapolis, MN, USA). Lipopolysaccharide isolated from *P. gingivalis* (PG-LPS) was purchased from Invivo Gen (San Diego, CA, USA). Mouse monoclonal antibodies iNOS and COX-2 secondary antibodies, anti-rabbit, anti mouse (Santa Cruz Biotechnology Inc., Dallas, TX, USA) were purchased from Cayman Chemical (Ann Arbor, MI, USA) for Western blot analysis. A Hybond ECL Nitrocellulose membrane (Amersham Pharmacia Biotech Inc., Piscataway, NJ, USA) Western blotting detection system was purchased from Advansta Inc. (Santa Clara, CA, USA). Alizarin Red S and tin protoporphyrin IX (SnPP) were obtained from Sigma-Aldrich (St. Louis, MO, USA).

### 2.2. Plant Materials

*P. ginseng* fruit was cultivated and harvested in Gyeonggi province Anseong (Anseong, Korea), and provided by the national agricultural cooperative federation of Gyeonggi province Anseong. The *P. ginseng* fruit extract was provided by Korea Ginseng Corporation (KGC) LIFENGIN INC. (Seoul, Korea) The preparation of *P. ginseng* fruit extract was enzymatically decomposed (amylase, pectinase, cellulose) at 50 °C for 4 h, which was followed by enzyme inactivation at 90 °C for 10 min. Then, it was prepared by filtering perlite and then vacuum concentration. The major components of *P. ginseng* fruit extract used in this study are shown in specified in the HPLC-DAD chromatogram of Figure S1.

### 2.3. Cell Culture

The protocols for the isolation and culture of HPDL (human periodontal ligment) cells were reviewed and approved by the Institutional Review Board of Kyungpook National University (Daegu, Korea) (KNU 2017-78). HPDL cells were obtained from the third molar of each donor as previously described [19]. The HPDL cells were cultured in α-MEM supplemented with 10% (*v*/*v*) fetal bovine serum (FBS) and 1% penicillin/streptomycin (Gibco BRL, Grand Island, NY, USA), and cultured at 37 °C in a humidified atmosphere with 5% CO_2_.

### 2.4. Cell Viability and Coefficient Assays

The HPDL cells (5 × 10^3^ cells/mL) were cultured in a 96-well plate with all concentrations of the PGFE for 24 h in a humidified atmosphere (37 °C, 5% CO_2_); then, 5 mg/mL of the MTT (3-[4,5-dimethylthiazol-2-yl]-2,5-diphenyl tetrazolium bromide) was added to 100 µL of cell suspension for 4 h. After the medium was removed, 200 µL of dimethyl sulfoxide (DMSO) was added. The spectrophotometric absorbance at 595 nm was measured using a microplate reader (Tecan Trading AG) (Männedorf, Switzerland), and for coefficient assays, cells were seeded in 24-well plates for 6 h with PGFE (50, 100, 150, 200 μg/mL) and then stimulated for 24 or 48 hr with PG-LPS (1 μg/mL). The cells were counted with Incucyte^®^ Live-Cell analysis systems (Göttingen, Germany).

### 2.5. Nitrite Assay Assays

The HPDL cells were treated with 50, 100, 150, and 200 μg/mL of PGFE and cultured for 6 h, then, PG-LPS was treated for 18 h. After the incubation, 100 μL of supernatant was added to 100 μL of Griess reagent (1% sulfanilamide, 2.5% phosphoric acid containing 0.1% naphthyl ethylene di-amine). After 10 min of reaction, the absorbance was measured at 540 nm. At this time, sodium nitrite (NaNO_2_) was prepared for each concentration to show a standard curve, and the NO production amount was calculated.

### 2.6. Cytokines Production Assay

The HPDL was cultured in a 96-well microplate at a density of 1 × 10^4^ cells/well and then treated with PGFE at 50, 100, 150, and 200 μg/mL for 6 h. Then, PG-LPS was treated for 18 h, the supernatant was taken, and the secretion amount of inflammatory cytokine, TNF-α, IL-6, or PGE2 (R & D system, Minneapolis, MN, USA) kit according to the manufacturer’s instructions.

### 2.7. Wound-Healing Assays

The HPDL cells were seeded into a 12-well culture plate (5 × 10^5^ cells/well) and cultured to confluence. A horizontal wound was scratched with a P-200 pipette tip in each well and then treated with PGFE (50, 100, 150, 200 μg/mL). The data for the scratched area were measured at 0, 24, and 48 h with a microscope.

### 2.8. Mineralization Assay

HPDL cells were cultured at 1 × 10^4^ cells/well in a 6-well culture plate and then prepared for osteogenic induction, containing 50 μg/mL ascorbic acid, 0.1 μM dexamethasone, and 10 mM β-glycerophosphate culture in osteo-induction medium (OIM) for 14 days. When mineralized nodules were formed, the mineralized cells were fixed with 4% polyformaldehyde for 30 min, stained with 0.1% Alizarin Red S (Sigma-Aldrich, St. Louis, MO, USA). at pH 4.3 for 30 min at room temperature, and washed with deionized water. The staining results were observed with a microscope (Nikon, Japan). To measure the content of calcium deposits, the cetyl pyridine chloride (CPC) method was applied, and absorbance was measured at 560 nm using a multifunctional microplate reader (M1000 Pro, TECAN, Männedorf, Switzerland).

### 2.9. Immunofluorescence Analysis

The cells were fixed in 4% paraformaldehyde for 20 min and incubated in Triton-X100 for 10 min to penetrate the cell membrane. Next, a goat serum blocking solution was added to each well for 1 h. Next, the cells were incubated with rabbit anti-Nrf2 antibody (Abcam, Cambridge, MA, USA; 1:100) overnight at 4 °C and further incubated with fluorescein isothiocyanate (FITC)-conjugated anti-rabbit IgG (Abcam, Cambridge, MA, USA; 1:1000) for 1 h at room temperature in the dark, and images were captured under a fluorescence microscope (LEICA, Wetzlar, Germany).

### 2.10. Cytosolic and Nuclear Protein Extraction

The HPDL cells were seeded at 5 × 10^5^ cells/mL in a 6-well plate. Then, the harvested cells were lysed on ice for 20 min with radioimmunoprecipitation assay (RIPA) buffer (Thermo Fisher Scientific, Waltham, MA, USA) and the isolated cytoplasm and nuclei were removed using the NE-PER nuclear and cytoplasmic extraction reagent kit (Pierce Biotechnology, Rockford, IL, USA) according to the manufacturer’s instructions.

### 2.11. Western Blot Analysis

The HPDL cells were treated with lysis buffer (50 mM Tris pH 8.0, 150 mM NaCl, 0.02% sodium azide, 0.2% SDS, 1 mM PMFS, 10 μL/mL aprotinin, 1% igapel 630 (Sigma-Aldrich, St. Louis, MO, USA), 10 mM NaF, and 0.5 mM EDTA), centrifuged at 23,000× *g* for 15 min, and the nuclear and cytoplasmic proteins were separated. The expression of NF-kB, a nuclear transcription factor, was measured using isolated nuclear proteins. Separated protein concentrations were measured and transferred to Hybond ECL Nitrocellulose membrane (Amersham Pharmacia Biotech Inc., Piscataway, NJ, USA) after loading in 20 μg of each well of SDS/8–12% polyacrylamide gel. The membrane is incubated at room temperature for 2 h with 5% (*w*/*v*) non-fat dried milk dissolved in tris buffer 10 mM Tris (pH 8.0) and 150 mM NaCl) containing 0.05%. Each membrane is incubated with primary antibody monoclonal antibodies overnight at 4 °C for 24 h at room temperature and washed with tris buffer. After this, anti-rabbit or anti-mouse (Santa Cruz Biotechnology Inc., Dallas, TX, USA) are incubated for 2 h with secondary antibody and washed with tris buffer. Proteins activated by binding to the antibody were measured using an ECL Western blotting detection system. Membranes were detected with Healthcare Life Science ECL-plus (Tokyo, Japan), and the images were taken by an ImageQuant LAS 4000 (GE Healthcare Life Science, Tokyo, Japan). The expressional value of cytosolic was normalized to the intensity level of β-actin using image J software (NIH, Stapleton, NY, USA).

### 2.12. RT-qPCR Analysis

The total RNA was extracted from the HPDL cells using TRIzol/chloroform reagent (Bioneer, Daejeon, Korea) according to the manufacturer’s instructions. Total RNA was transcribed into cDNA by a PrimeScript-RT reagent kit; then, the cDNA was amplificated by the SYBR Premix Ex Taq (Sangon). The cycling conditions were 40 cycles at 50 °C for 2 min, 95 °C initial denaturation for 10 min, 95 °C denaturation for 15 s, and 60 °C annealing for 30 s. The mRNA encoding each target was measured using real-time PCR, and GAPDH was used as the housekeeping gene. The cycle threshold (Ct) value of the target gene was normalized to GAPDH. The primers for each gene used in this study were supplied by Biomedic Co, (Bucheon, Korea) and the sequence of primers is shown in Table 1.

### 2.13. Animal

Male 8-week-old C57BL/6 mice mice and 9-week-old Sprague–Dawley rats were purchased from Samtako Inc. (Osan, Korea). Experiments with rats were conducted in the animal laboratory of College of Pharmacy Keimyung University (Daegu, Korea), and experiments with mice were conducted at School of Dentistry Kyungpook National University (Daegu, Korea). The entire experimental procedure was authorized by the Institutional Animal Care and Use Committee (IACUC) of the Keimyung University Laboratory Animal Research Center (Permit No. KM2020-005).

### 2.14. Ligature-Induced Periodontitis Model

Male C57BL/6 mice (8 weeks old, *n* = 2 per group, 12 in total) were ligated with non-absorbable braided silk for 5 days around the second molars. After 5 days, the ligation was removed along with the upper second molar, and 50 μg of PGFE was treated for 7 and 14 days, respectively. The mice with ligature were sacrificed to harvest maxilla with an intact palate. Harvested maxillae were dissected to separate the left experimental and right control half hemi-maxillae, which were autoclaved in phosphate-buffered saline for 15 min. After defleshing, hemi-maxillae were stained with 1% methylene blue (CAS: 7220-79-3, Sigma-Aldrich, St. Louis, MO, USA) for 1 min, and the stained maxillae were washed with distilled water, the images of bone loss and alveolar bone were captured using 2D/3D-micro CT by staining methylene blue.

### 2.15. PG-LPS-Induced Periodontitis Model

The Sprague–Dawley rat group was divided into 5 groups 1 week after adaptation: (1) CON (PBS injection); (2) PG-LPS (6 day induced); (3) PG-LPS (6 day induced) + PGFE50 (50 mg/kg for 8 days); (4) PG-LPS (6 day induced) + PGFE100 (100 mg/kg for 8 days); and (5) PG-LPS (6 day induced) + PGFE200 (200 mg/kg for 8 days). Periodontitis was induced in all the groups except for the control group by injecting 10 mg/mL of PG-LPS every day between the first and second mandibular molars using isoflurane (Hana Pharm. Co., Ltd., Gyeonggido, Korea) as an anesthetic. After the experiment was completed, the maxillary skull was removed by sacrificing the experimental animal, and the experiment method was the same as in Section 2.13.

### 2.16. Micro-CT Imaging and Analysis

Micro CT (Quantum FX micro-CT, Perkin Elmer, Waltham, MA, USA) were scanned at tube voltage (90 kVp), tube current (160 μA), imaging time (180 sec), FOV (field of view, 5 mm), and Pixil size (10 μM). To measure bone mineral density (BMD), the direction was changed to a coronal section, and the raw data obtained from Micro-CT were loaded into CTAn and scanned images, bone mineral density (BMD) were obtained based on the above threshold. The region of interest (ROI) was set using the interpolation method of the alveolar bone except the root of the cement–enamel junction to the root. Data were expressed as mean ± SD. The significance of the data was analyzed by one-way ANOVA using SPSS Statistics (Armonk, NY, USA).

### 2.17. Histological Staining

For hematoxylin and eosin (H&E) staining, the periodontal tissue removed from the maxillary skull was fixed with 10% formalin, and then embedded in paraffin, cut into 5 μm sections, and fixed on slides. The tissue fixed on the slide was stained with H&E. After then, the tissue infiltration was observed using a fluorescence Olympus IX microscope 71-F3 2PH (Tokyo, Japan).

### 2.18. Statistical Analysis

Each experiment was performed in triplicate and expressed as a mean value and standard deviation. Statistical analysis was conducted using SPSS Statistics 19.0 software (Armonk, NY, USA). Differences among groups were analyzed by one-way analysis of variance (ANOVA) followed by Tukey’s test or Student’s *t*-test. *p* < 0.05 were considered to indicate statistical significance.

## 3. Results

### 3.1. P. ginseng Fruit Extract (PGFE) Is Not Cytotoxic and Promotes HPDL Cell Proliferation

**Figure 1:**
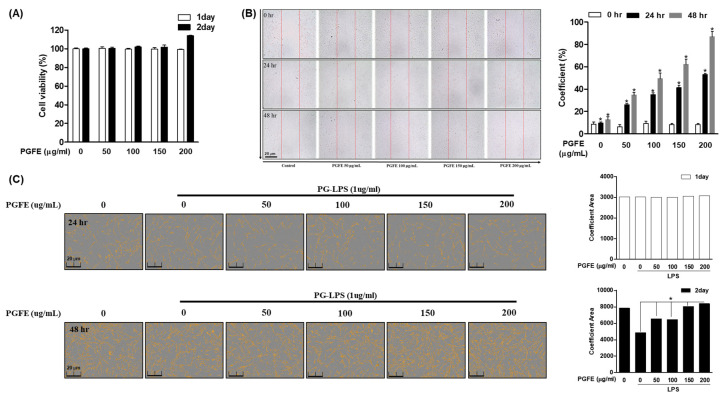
*Panax ginseng* fruit extract (PGFE) is not cytotoxic and showed a cell proliferation effect on HPDL cells. (**A**) HPDL cells were seeded at a density of 1 × 10^4^ cells/well and treated with PGFE at the indicated concentrations (0–200 μg/mL) for 24 or 48 hr; then, afterwards, PGFE cytotoxicity was investgated by 3-(4,5-dimethylthiazol-2-yl)-2,5-diphenyltetrazoliumbromide (MTT) assay. (**B**) HPDL cells at a concentration of 5 × 10^5^ cells/mL were seeded on a 6-well plate, scratched at regular intervals, treated with PGFE at the indicated concentration, and observed for 48 hr to measure the cell coefficient of the interval of scratches recovered by the cells. (**C**) The confluency of cells was determined using an IncuCyte imaging system. The Student’s *t*-test was used for statistical analysis. Differences were considered to be significant at values of * *p* < 0.05. Scale bars denoted 20 μm.

To investigate whether PGFE (Figure 1A) exerts a cytotoxic effect on HPDL cells, MTT assay was performed using PGFE at concentrations of 0, 50, 100, 150, and 200 μg/mL for 24 or 48 h. The indicated concentrations of PGFE did not show cytotoxicity. Moreover, treatment with PGFE at 200 μg/mL for 48 h promoted HPDL cell proliferation. Therefore, to investigate the proliferative effect of PGFE on HPDL cells, wound healing (Figure 1B) and cell coefficient assays (Figure 1C) were performed for 48 h after treatment at a concentration of 50–200 μg/mL. After 48 h of treatment with PGFE, the scratch wound made on the cells recovered in a dose-dependent manner, and the cell coefficient increased.

### 3.2. PGFE Promotes HO-1 Expression and Nrf2 Translocation in HPDL Cells

**Figure 2:**
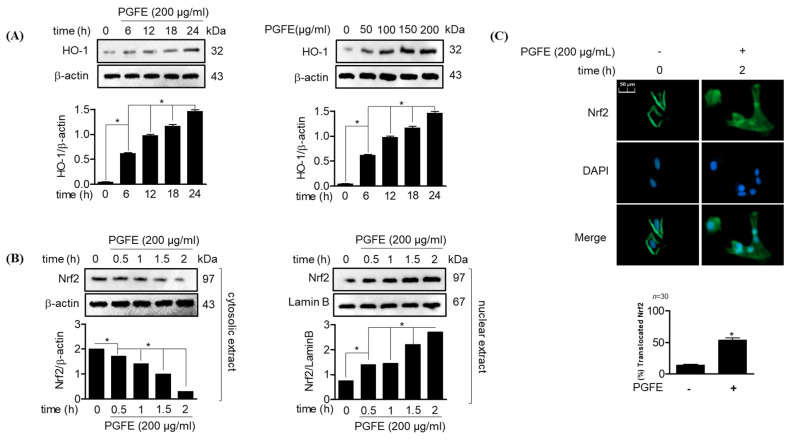
Effects of PGFE on heme oxygenase (HO)-1 induction and the promotion of nuclear factor-erythroid 2-related factor 2 (Nrf2) in HPDL cells. (**A**) The cells (1 × 10^6^ cells/mL) were treated with 200 μg/mL for the indicated time (0, 6, 12, 18, 24 hr) or with the indicated concentrations of PGFE (50, 100, 150, and 200 μg/mL) for 24 hr. (**B**) The translocation of Nrf2 was analyzed by Western blot analysis from cells treated with 200 μg/mL of PGFE. (**C**) After treatment with the indicated concentration of PGFE within 2 hr, Nrf2 immunostaining was performed to confirm translocation. * *p* < 0.05 vs. each treated group. Scale bars denoted 50 μm.

The induction of Nrf2 and HO-1 expression is known to play an important role in controlling oxidative stress and inhibiting inflammatory responses. Therefore, the effect of PGFE on the translocation of the major transcription factor Nrf2, which regulates the protein and gene expression of HO-1, was investigated through Western blotting following cell treatment with PGFE for 6, 12, 18, and 24 h. The results showed that HO-1 was expressed at 6 h after PGFE treatment, and the expression was the highest at 24 h. PGFE increased the expression of HO-1 in a concentration-dependent manner at the concentrations of 50–200 μg/mL (Figure 2A). The accumulation of Nrf2 in the nucleus and cytoplasmic fractions of HPDL cells after PGFE treatment confirmed the effect of PGFE on the translocation of Nrf2. Moreover, within 2 h of PGFE treatment, the Nrf2 level in the cytoplasmic fraction decreased and Nrf2 accumulated in the nucleus (Figure 2B). In addition, immunofluorescence assay result revealed that the effect of PGFE on Nrf2 translocation was time dependent, confirming the results of the Western blotting assay (Figure 2C). These results suggested that PGFE promotes HO-1 expression by inducing Nrf2 translocation in HPDL cells.

### 3.3. Inhibitory Effects of PGFE on PG-LPS-Induced Expression of Pro-Inflammatory Cytokines and Mediators in HPDL Cells

**Figure 3:**
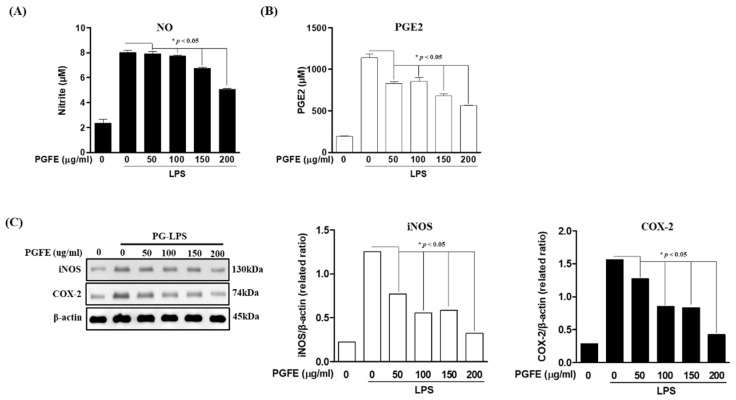
Inhibitory effects of PGFE on *Porphyromonas gingivalis* lipopolysaccharide (PG-LPS) induced pro-inflammatory mediators in HPDL cells. The HPDL cells were pretreated with the indicated concentrations (50–200 μg/mL) of PGFE for 6 h and then stimulated with or without PG-LPS for 24 h. The level of (**A**) NO and (**B**) PGE2 production was measured by an ELISA kit. (**C**) The expression of inducible nitric oxide synthase (iNOS) and cyclooxygenase-2 (COX-2) proteins were measured by Western blot analysis from cells pretreated with PGFE and stimulated by PG-LPS. * *p* < 0.05 was considered significant compared to only the PG-LPS treated group.

**Figure 4:**
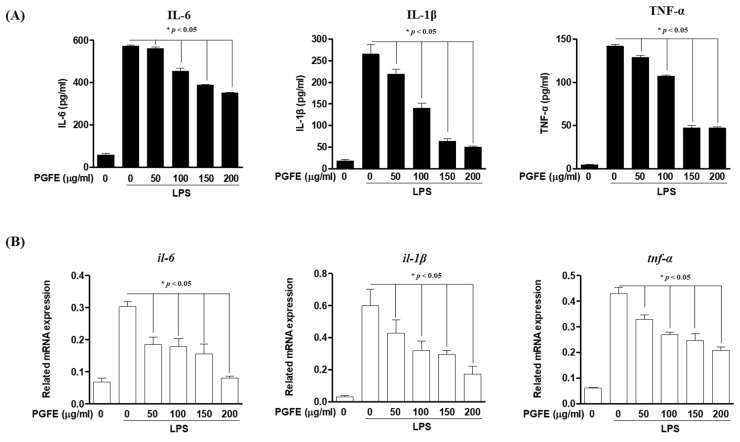
Inhibitory effects of PGFE on PG-LPS induced pro-inflammatory cytokines in HPDL cells. The HPDL cells were pretreated with the indicated concentrations (50–200 μg/mL) of PGFE for 6 h and then stimulated with or without PG-LPS for 24 h. (**A**) The expression of pro-inflammatory cytokines production was measured by an ELISA kit. (**B**) The level of pro-inflammatory cytokines mRNA were measured by real-time PCR analysis. * *p* < 0.05 was considered significant compared to only the PG-LPS treated group.

To investigate the anti-inflammatory effect of PGFE in HPDL cells, the effect of PGFE on the expression of pro-inflammatory cytokines and mediators induced by *Porphyromonas gingivalis* lipopolysaccharide (PG-LPS) (1 μg/mL) was evaluated. The protein level of NO, PGE2, and the pro-inflammatory mediator iNOS and COX-2 in the medium supernatants of HPDL cells was measured after pretreatment with PGFE (50–200 μg/mL) for 6 h and subsequent treatment with PG-LPS for 24 h. The results confirmed that PGFE, in a concentration-dependent manner, inhibited the production of NO (Figure 3A) and PGE2 (Figure 3B) as well as decreased the PG-LPS-induced increase in iNOS and COX-2 protein levels (Figure 3C). In addition, PGFE effectively regulated the expression of the pro-inflammatory cytokines IL-6, IL-1β, and TNF-α, which play an important role in the regulation of periodontitis in PG-LPS-stimulated HPDL cells (Figure 4A), and it effectively suppressed the mRNA levels of these pro-inflammatory cytokines (Figure 4B). These results suggested that PGFE can inhibit periodontitis in an in vitro model of periodontitis induced by PG-LPS.

### 3.4. PGFE Inhibits PG-LPS-Induced Pro-Inflammatory Cytokines by Promoting HO-1 Expression

**Figure 5:**
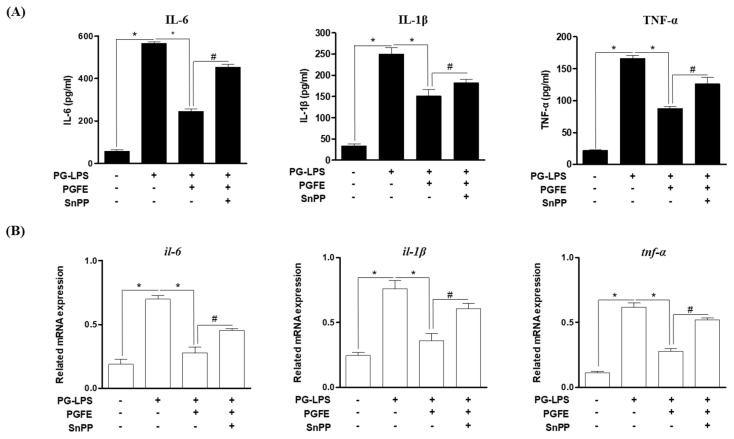
Inhibitory Effect of PGFE through by HO-1 on PG-LPS induced pro-inflammatory cytokines. The HPDL cells were pretreated with the indicated concentration of PGFE (200 μg/mL) for 6 hr and then incubated with PG-LPS for 24 hr. (**A**) The production of pro-inflammatory cytokines interleukin (IL)-6, IL-1β and tumor necrosis factor-α (TNF-α) were determined by ELISA kit assay. (**B**) The mRNA levels of pro-inflammatory cytokines genes and *il-6*, *il-1β,* and *TNF-α* genes were determined by real-time PCR analysis. The results were normalized to *gapdh* expression. * *p* < 0.05 vs. only LPS treated group; ^#^ *p* < 0.05 vs. LPS + PGFE treated group.

We evaluated whether the effect of PGFE on HO-1 expression and Nrf2 translocation, as shown in Section 3.2, affects the expression of the pro-inflammatory cytokines involved in periodontitis. HPDL cells were pretreated with PGFE (200 μg/mL) for 6 h in the presence or absence of SnPP (20 μM), which is a competitive inhibitor of HO-1, and then treated with LPS (1 μg/mL) for 24 h. The results showed that the anti-inflammatory effect of PGFE was reversed by treatment with SnPP (Figure 5A). In addition, the mRNA levels of pro-inflammatory cytokines were evaluated to further confirm this finding. The result revealed that the LPS-induced increases in the mRNA levels of IL-6, IL-1β, and TNF-α were decreased by PGFE (200 μg/mL) and that SnPP decreased these mRNA levels. These results were similar to the production of pro-inflammatory cytokines (Figure 5B). Thus, in an in vitro model of periodontitis induced by PG-LPS, PGFE may activate the Nrf2/HO-1 signaling pathway and suppress the expression of pro-inflammatory cytokines, resulting in anti-inflammatory effects.

### 3.5. Osteogenic Induction of HPDL Cells by PGFE

**Figure 6:**
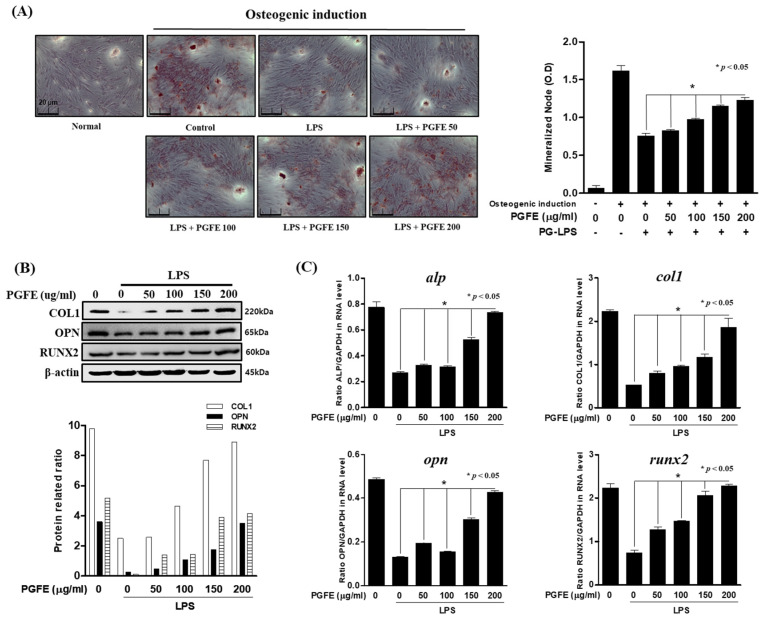
The effect of PGFE on osteogenic induction in HPDL cells. (**A**) The periodontal ligament (PDL) cells were pretreated with 50 100, 150 and 200 μg/mL for 6 h and then incubated with LPS for 7 days. (**B**) The protein expressions were confirmed by Western blot analysis. (**C**) The mRNA levels of *alp*, *col1, opn,* and *runx2* were measured by real-time PCR. The results were normalized to *gapdh* or β-actin expression. * *p* < 0.05 was considered significant compared to only the PG-LPS treated group. Scale bars denoted 20 μm.

Suppression of the inflammatory response and recovery of alveolar bone loss due to the disease are strategies to treat periodontitis. Therefore, in an in vitro model of periodontitis induced by PG-LPS, the inhibitory effect of PGFE on periodontal inflammation and alveolar bone loss was evaluated. To investigate the mRNA and protein levels of osteoblast markers in the early stages of osteoblast differentiation, we induced osteoblast differentiation for 7 days using α-MEM containing β-glycerophosphate, ascorbate, and dexamethasone. The effect of PGFE on osteoblast differentiation was evaluated by comparing the group treated with PG-LPS alone with the group treated with PG-LPS and PGFE. Alizarin Red S staining showed that HPDL cells were successfully differentiated into osteoblasts, and PGFE reversed the osteoblast differentiation inhibited by PG-LPS in a concentration-dependent manner (Figure 6A). In addition, PGFE recovered protein (Figure 6B) and mRNA (Figure 6C) levels of the osteoblast differentiation markers ALP, COL1, OPN, and RUNX2 in a concentration-dependent manner.

### 3.6. Effect of PGFE-Induced HO-1 Expression on Osteogenic Differentiation

**Figure 7:**
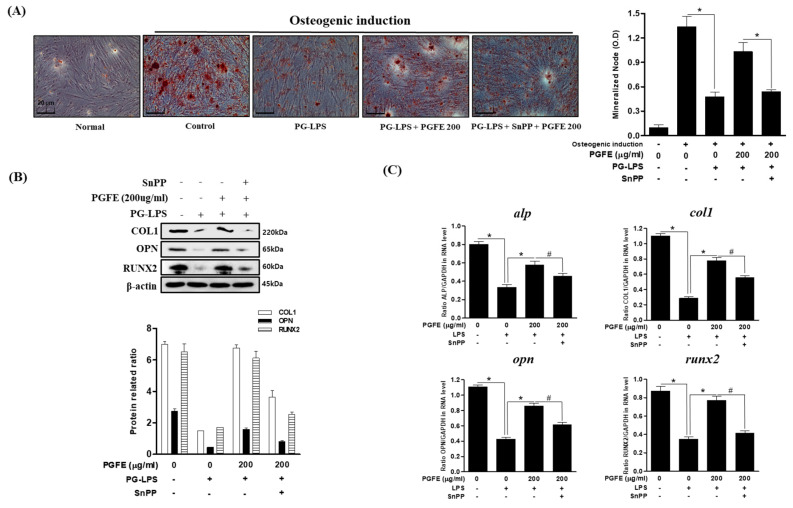
Effect of HO-1 expression by PGFE on osteogenic induction. (**A**) The PDL cells were pretreated with or without the indicated concentration of tin protoporphyrin IX (SnPP), then after, PGFE (200 μg/mL) for 6 h and then incubated with lipopolysaccharides (LPS) for 7 days. (**B**) The protein expressions were confirmed by Western blot analysis. (**C**) The mRNA levels of *alp*, *col1, opn,* and *runx2* were measured by real-time PCR. The results were normalized to *gapdh* or β-actin expression. * *p* < 0.05 vs. only LPS treated group; ^#^ *p* < 0.05 vs. LPS + PGFE treated group. Scale bars denoted 20 μm.

As described in Section 3.5, PGFE restored the differentiation of PDL cells into osteoblasts, which was inhibited by PG-LPS. Therefore, the effect of HO-1 expression induced by PGFE on osteoblast differentiation was evaluated. In an in vitro model of PG-LPS-induced periodontitis, the mRNA and protein levels of osteoblast differentiation markers were evaluated following treatment with SnPP. First, cells were pretreated with SnPP for 2 h and then treated with PGFE and PG-LPS via the same methods described in Section 3.5 to induce osteogenic differentiation for 7 days in α-MEM containing β-glycerophosphate, ascorbate, and dexamethasone. The results confirmed that the differentiation of HPDL cells into osteoblasts was inhibited in the PG-LPS-only group, but this was recovered in the PG-LPS + PGFE group. In addition, osteogenic differentiation was significantly suppressed by SnPP compared with that by PGFE only (Figure 7A). Moreover, the protein (Figure 7B) and mRNA (Figure 7C) levels of ALP, COL1, OPN, and RUNX2 were decreased. These results suggested that the promotion of HO-1 expression by PGFE is involved in the osteogenic induction and the anti-inflammatory effects of PGFE in HPDL cells under periodontitis conditions.

### 3.7. Inhibitory Effect of PGFE on Periodontitis in a Ligature-Induced In Vivo Model

**Figure 8:**
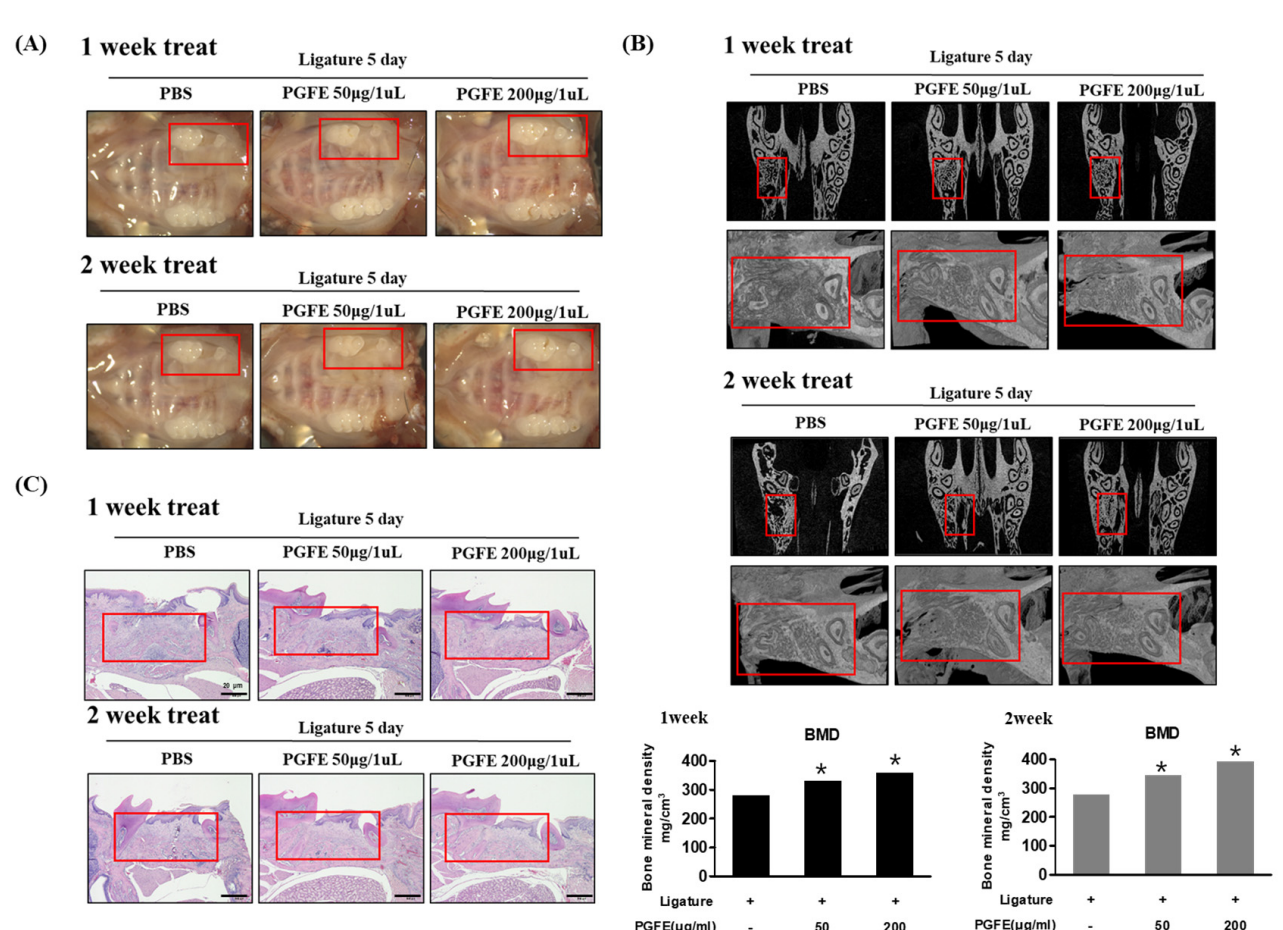
Inhibitory effect of PGFE on periodontitis in a ligature-induced in vivo model. (**A**) Induction of periodontitis; microscopic photographs of PGFE at the indicated concentration in the extraction socket (red area) of the second molars were treated for 1 or 2 weeks. (**B**) Micro-CT (computed tomography) analysis of newly formed bone by extraction socket (red area) and PGFE. Analysis tables were determined using CTAn software. (**C**) Histological analysis of the periodontium using hematoxylin and eosin (H&E) staining. * *p* < 0.05 vs. only phosphate-buffered saline treated group. Scale bars denoted 20 μm.

In vitro studies confirmed the effect of PGFE in inhibiting periodontitis and inducing osteogenic differentiation. Therefore, the effect of PGFE on periodontitis in vivo was evaluated in mouse models of ligature-induced periodontitis, which is a representative model of the disease. The induction site was treated with PGFE for 1 or 2 weeks. To induce periodontitis, the left upper second molar was tied and maintained with silk for 5 days. Next, the second molar was extracted, and the socket of the extracted molar region was treated with phosphate-buffered saline (PBS) or PGFE (50 and 200 μg/mL) for 1 or 2 weeks. Finally, the maxillary skull was photographed. The results confirmed that the loss of the periodontal socket via ligature was recovered after 1 and 2 weeks of PGFE treatment (Figure 8A). In addition, micro-computed tomography (CT) of the bones in the root region of the socket after tooth extraction showed that PGFE increased the alveolar bone volume (PBS group: week 1/2, 280.3/276.2 mg/cm^3^; PGFE 50 μg/mL group: week 1/2, 329.9/343.4 mg/cm^3^; PGFE 200 μg/mL group: week 1/2, 357.0/392.4 mg/cm^3^) and bone mineral density of the tooth socket (Figure 8B). In addition, the effect of PGFE on cell infiltration due to periodontitis was evaluated by hematoxylin and eosin staining. Compared with the PBS group, which showed cell infiltration, the 50 and 200 μg/mL PGFE groups showed a concentration-dependent attenuation of cell infiltration and irregular surface formation (Figure 8C). These results suggested that PGFE inhibited periodontitis and restored alveolar bone in an in vivo model of periodontitis.

### 3.8. Inhibitory Effect of PGFE on Periodontitis in PG-LPS Induced Periodontitis In Vivo Model

**Figure 9:**
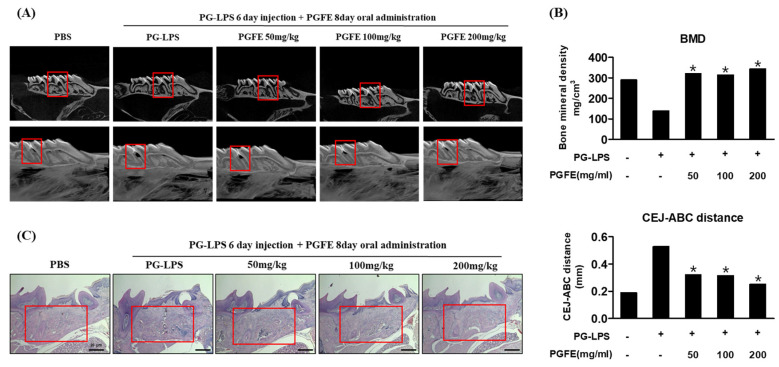
Inhibitory effect of PGFE on periodontitis in PG-LPS-induced in vivo model. (**A**) Micro-CT analysis of newly formed bone by PGFE, the red mark is the PG-LPS injection area. (**B**) Quantification of distance between CEJ and ABC, and bone mineral density. Analysis tables were determined using CTAn software. (**C**) Histological analysis of the periodontium using H&E staining. * *p* < 0.05 vs. only PG-LPS treated group. Scale bars denoted 20 μm.

To support the results described in Section 3.7, the efficacy of PGFE was verified in an in vivo model of PG-LPS-induced periodontitis. To confirm the efficacy of PGFE in inhibiting periodontitis and inducing bone formation in vivo, PG-LPS was injected between the first and second molar teeth in the right maxilla of 9-week-old Sprague–Dawley rats for 6 days, followed by the oral administration of PGFE for 8 days. The extent of alveolar bone loss was determined using micro-CT. Alveolar bone loss between the first and second molars was observed in rats treated with PG-LPS. However, PGFE suppressed the alveolar bone loss in a concentration-dependent manner (Figure 9A). In addition, bone mineral density loss caused by PG-LPS was restored by PGFE, as revealed by measuring the extent of alveolar bone loss from the cementoenamel junction to the alveolar bone crest. The extent of alveolar bone loss following PG-LPS treatment was 0.53 mm, but after treatment with 50, 100, and 200 μg/mL, PGFE was only 0.32, 0.31, and 0.25 mm, respectively, showing a dose-dependent decrease (Figure 9B). In the PG-LPS-induced periodontitis model, PGFE also inhibited the inflammatory invasion of periodontal cells in a concentration-dependent manner (Figure 9C). These results showed that PGFE suppressed periodontitis and restored alveolar bone loss in various in vivo periodontitis models, supporting the results of the in vitro studies.

## 4. Discussion

The pathogenesis of periodontitis begins with the release of endotoxins by bacteria. Next, pro-inflammatory factors, such as TNF-α, IL-1β, and IL-6, are secreted from local PDL cells and invade the periodontal tissue. Eventually, periodontitis is a chronic infectious disease that is absorbed and destroyed [20]. PDL cells, which are abundant in periodontal tissues, maintain the homeostasis and physiological function of periodontal structures; these cells also have the potential for multidirectional differentiation into fibroblasts, osteoblasts, or cementum cells [21,22,23]. Therefore, the treatment strategy for periodontitis is to suppress the secretion of pro-inflammatory factors as well as the absorption and destruction of the periodontium and PDL cells. The expression of HO-1, an important cytoprotective enzyme, indicates an adaptative and protective response to LPS stress in various cells [24]. In previous studies, PG-LPS was shown to be a major pathogenic factor of chronic periodontitis that stimulates PDL cells to produce pro-inflammatory factors, such as TNF-α and IL-1β, causing the destruction of the alveolar bone [25]. In this study, we investigated whether PGFE affects the expression of pro-inflammatory factors and induces osteogenic differentiation by regulating HO-1 expression in human PDL cells stimulated with PG-LPS. Moreover, we verified its efficacy using an animal model of periodontitis induced by ligature and PG-LPS. The results of this study confirmed that PGFE induced cell proliferation in an in vitro human PDL cell model of PG-LPS-induced periodontitis and regulated HO-1 expression by inducing the transcription of Nrf-2 to the nucleus.

According to a previous study, HO-1 upregulation incudes an anti-inflammatory effect that is mediated by inhibiting the release and production of pro-inflammatory cytokines and pro-inflammatory mediators, such as iNOS and COX-2. In addition, it induces the osteoblast differentiation of MC3T3-E1 cells [26,27]. In this study, PGFE inhibited the production of TNF-α, IL-1β, and IL-6, which are the major cytokines involved in periodontitis induced by PG-LPS, as well as the pro-inflammatory mediators NO and PGE2. A study assessing SnPP, an inhibitor of HO-1, revealed that the anti-inflammatory effect of PGFE was mediated via the expression of HO-1. In addition, alveolar bone resorption is the most important pathological feature of periodontitis. In an inflammatory environment, periodontal homeostasis cannot balance these processes, resulting in alveolar bone resorption and tooth loss [28,29]. By promoting HO-1 expression, PGFE recovered PG-LPS-induced decreases in the protein and mRNA levels of the bone morphogenic transcription factors alkaline phosphatase (ALP), collagen type I (COL1), osteopontin (OPN), and runt-related transcription factor 2 (Runx2), which are important for bone formation and differentiation, in human PDL cells.

The *P. ginseng* fruit extract used in this study was obtained through enzymatic decomposition processes such as amylase, pectinase, and cellulase. According to previous reports, this enzymatic decomposition minimizes the destruction of major components more so than hot water extraction, and it has been reported that it can increase the extraction yield of useful components [30,31]. In this study, using this *P. ginseng* fruit extract, we investigated the inhibitory effect of PGFE on a periodontitis model using the ligature method and induced PG-LPS, which are representative models most widely used in periodontitis research [32,33]. Of the two models, the ligature induction method was a more severe model than the periodontitis model induced by PG-LPS, and the PGFE showed an inhibitory effect on periodontitis induced by the PG-LPS and ligature methods. These results suggest that PGFE has an inhibitory effect on periodontitis in both oral administration and local treatment methods, so both treatment methods of PGFE are considered to be meaningful treatments for periodontitis. Therefore, the in vitro results were verified in an animal model of periodontitis, in which PGFE was confirmed to restore the alveolar bone loss caused by ligature and PG-LPS, to recover the level of bone mineral density (an important indicator for bone formation), and to improve inflammation-induced infiltration of cells in periodontal tissues. These effects of PGFE on periodontitis were similar to those reported previously [34,35], supporting its in vitro efficacy. Irrespective, this study has a limitation. The role of HO-1 in the inhibitory effect of PGFE on periodontitis was not evaluated in the in vitro model, but the in vitro study showed the anti-inflammatory effects and osteogenic induction by PGFE through HO-1 expression. Therefore, it is believed that the inhibitory effect of PGFE on periodontitis in vivo is related to the expression of HO-1. Further studies will be conducted in the future to confirm this.

## 5. Conclusions

In this study, PGFE inhibited the production and expression of pro-inflammatory cytokines and pro-inflammatory mediators as well as increased osteogenic induction by regulating the expression of HO-1 in human PDL cells stimulated with PG-LPS. In addition, this efficacy was demonstrated in an in vivo animal model. Therefore, PGFE has potential as a new therapeutic material for periodontal diseases that acts by inhibiting inflammation, which is an important target for the treatment of periodontitis and recovery of alveolar bone loss caused by inflammation.

## Figures and Tables

**Table 1:** Primer sequences.

Target Gene	Sequence (5′→3′)		Accession Number
* il-6 *	Forward	AGTGAGGAACAAGCCAGAGC	NM_000600.4
	Reverse	GTCAGGGGTGGTTATTGCAT	
* il-1β *	Forward	AACCTCTTCGAGGCACAAGG	NM_000576.2
	Reverse	GTCCTGGAAGGAGCACTTCAT	
* tnf-α *	Forward	GCCTCTTCTCCTTCCTGATCGT	NM_000594.2
	Reverse	TGAGGGTTTGCTACAACATGGG	
* alp *	Forward	TGCAGTACGAGCTGAACAGG	NM_000478
	Reverse	GTCAATTCTGCCTCCTTCCA	
* col1 *	Forward	CCAGAAGAACTGGTACATCAGCAA	NM_000088
	Reverse	CGCCATACTCGAACTGGAATC	
* opn *	Forward	TCAGCTGGATGACCAGAGTG	NM_001040060
	Reverse	TTGGGGTCTACAACCAGCAT	
* runx2 *	Forward	TCTTAGAACAAATTCTGCCCTTT	NM_001024630.3
	Reverse	TGCTTTGGTCTTGAAATCACA	
* gapdh *	Forward	TGTTCGTCATGGGTGTGAAC	NM_002046
	Reverse	GTCTTCTGGGTGGCAGTGAT	

## Supplementary Materials

The following are available online at https://www.mdpi.com/2076-3921/9/12/1221/s1, Figure S1: The chromatograms of the *P. ginseng* fruit extract (A) and standard compound solutions G-Re (B), G-Ra8 (C), and G-Rf (D) obtained using HPLC-DAD.

## References

[1] Profio B.D., Villar C.C., Saraiva L., Ortega L.K., Pannuti C.M. (2017). Is periodontitis a risk factor for infections in cirrhotic patients. Med. Hypotheses.

[2] Genco R.J. (1996). Current view of risk factors for periodontal diseases. J. Periodontol..

[3] Pihlstrom B.L., Michalowicz B.S., Johnson N.W. (2005). Periodontal diseases. Lancet.

[4] Guha M., Mackman N. (2001). LPS induction of gene expression in human monocytes. Cell. Signal..

[5] Kjeldsen M., Holmstrup P.F., Bendtzen K. (1993). Marginal Periodontitis and Cytokines: A Review of the Literature. J. Periodontol..

[6] Maruyama T., Tomofuji T., Endo Y., Irie K. (2011). Supplementation of green tea catechins in dentifrices suppresses gingival oxidative stress and periodontal inflammation. Arch. Oral Biol..

[7] Shao M.Y., Huang P., Cheng R., Hu T. (2009). Interleukin-6 polymorphisms modify the risk of periodontitis: A systematic review and meta-analysis. J. Zhejiang Univ. Sci. B.

[8] Hayami T., Zhang Q., Kapila Y., Kapila S. (2007). Dexamethasone’s enhancement of osteoblastic markers in human periodontal ligament cells is associated with inhibition of collagenase expression. Bone.

[9] Bao T.H.L., Doan V.N., Le H.T.N., Ngo L.T.Q. (2014). Various methods for isolation of multipotent human periodontal ligament cells for regenerative medicine. In Vitro Cell. Dev. Biol. Anim..

[10] Ari G., Cherukuri S., Namasivayam A. (2016). Epigenetics and Periodontitis: A Contemporary Review. J. Clin. Diagn. Res..

[11] Bezerra M.M., Lima V., Alencar V.B., Vieira I.B., Brito G.A., Ribeiro R.A., Rocha F.A. (2000). Selective cyclooxygenase-2 inhibition prevents alveolar bone loss in experimental periodontitis in rats. J. Periodontol..

[12] Otterbein L.E., Choi A.M. (2000). Heme oxygenase: Colors of defense against cellular stress. Am. J. Physiol. Lung Cell. Mol. Physiol..

[13] Zhang H., Davies K.J.A., Forman H.J. (2015). Oxidative stress response and Nrf2 signaling in aging. Free Radic. Biol. Med..

[14] Lee D., Kook S.H., Ji H., Lee S.A., Choi K.C., Lee K.Y., Lee J.C. (2015). N-acetyl cysteine inhibits H_2_O_2_-mediated reduction in the mineralization of MC3T3-E1 cells by down-regulating Nrf2/HO-1 pathway. BMB Rep..

[15] Choi E.M., Suh K.S., Kim Y.J., Hong S.M., Park S.Y., Chon S. (2016). Glabridin alleviates the toxic effects of Methylglyoxal on osteoblastic MC3T3-E1 cells by increasing expression of the glyoxalase system and Nrf2/HO-1 signaling and protecting mitochondrial function. J. Agric. Food Chem..

[16] Park H.J., Kim D.H., Park S.J., Kim J.M., Ryu J.H. (2012). Ginseng in traditional herbal prescriptions. J. Ginseng Res..

[17] Zhao G.R., Xiang Z.J., Ye T.X., Yuan Y.J., Guo Z.X. (2006). Antioxidant activities of Salvia miltiorrhiza and *Panax notoginseng*. Food Chem..

[18] Lee I.A., Hyam S.R., Jang S.E., Han M.J., Kim D.H. (2012). Ginsenoside Re ameliorates inflammation by inhibiting the binding of lipopolysaccharide to TLR4 on macrophages. J. Agric. Food Chem..

[19] Seo B.M., Miura M., Gronthos S., Bartold P.M., Batouli S., Brahim J., Young M., Robey P.G., Wang C.Y., Shi S. (2004). Investigation of multipotent postnatal stem cells from human periodontal ligament. Lancet.

[20] Assuma R., Oates T., Cochran D., Amar S., Graves D.T. (1998). IL-1 and TNF antagonists inhibit the inflammatory response and bone loss in experimental periodontitis. J. Immunol..

[21] Zhu W., Liang M. (2015). Periodontal ligament stem cells: Current status, concerns, and future prospects. Stem Cells Int..

[22] Bassir S.H., Wisitrasameewong W., Raanan J., Ghaffarigarakani S., Chung J., Freire M., Andrada L.C., Intini G. (2016). Potential for stem cell-based periodontal therapy. J. Cell. Physiol..

[23] Nomura Y., Yashiro Y., Sanggarnjanavanich S., Yamaguchi T., Arai C., Noda K., Takano Y., Nakamura Y., Hanada N. (2012). Human periodontal ligament fibroblasts are the optimal cell source for induced pluripotent stem cells. Histochem. Cell Biol..

[24] Otterbein L.E., Soares M.P., Yamashita K., Bach F.H. (2003). Heme oxygenase-1: Unleashing the protective properties of heme. Trends Immunol..

[25] Page R.C. (1991). The role of inflammatory mediators in the pathogenesis of periodontal disease. J. Periodontal. Res..

[26] Jeong G.S., Lee D.S., Li B., Kim J.J., Kim E.C., Kim Y.C. (2011). Anti-inflammatory effects of lindenenyl acetate via heme oxygenase-1 and AMPK in human periodontal ligament cells. Eur. J. Pharmacol..

[27] Takanche J.S., Kim J.E., Han S.H., Yi H.K. (2020). Effect of gomisin A on osteoblast differentiation in high glucose-mediated oxidative stress. Phytomedicine.

[28] Ram V.S., Sudhakar U.P. (2015). Bone biomarkers in periodontal disease: A review article. J. Clin. Diagn. Res..

[29] Enomoto H., Furuichi T., Zanma A. (2004). Runx2 deficiency in chondrocytes causes adipogenic changes in vitro. J. Cell Sci..

[30] Im G.Y., Ma J.Y., Kim K.W., Choi J.K., Kang D.K., Kwon T.R., Jang S.Y., Jeong Y.J. (2011). Quality characteristics of 4 year-old ginseng by enzymatic hydrolysis conditions. J. Korean Soc. Food Sci. Nutr..

[31] Kim N.M., Lee J.S., Lee B.H. (2000). Enzymatic hydrolysis of korean ginseng starch and characteristics of produced maltooligosaccharides. J. Ginseng Res..

[32] Kook K.E., Kim C., Kang W., Hwang J.K. (2018). Inhibitory Effect of Standardized *Curcuma xanthorrhiza* Supercritical Extract on LPS-Induced Periodontitis in Rats. J. Microbiol. Biotechnol..

[33] Kim Y.G., Kim M.O., Kim S.H., Kim H.J., Pokhrel N.K., Lee J.H., Lee H.J., Kim J.Y., Lee Y. (2020). 6-Shogaol, an active ingredient of ginger, inhibits osteoclastogenesis and alveolar bone resorption in ligature-induced periodontitis in mice. J. Periodontol..

[34] Choi G.E., Hyun K.Y. (2020). Inhibitory effect of Acer tegmentosum maxim extracts on *P. gingivalis* LPS induced Periodontitis. Arch. Oral Biol..

[35] Lee B.A., Lee H.S., Jung Y.S., Kim S.W., Lee Y.W., Chang S.H., Chung H.J., Kim O.S., Kim Y.J. (2013). The Effects of a Novel Botanical Agent on Lipopolysaccharide-Induced Alveolar Bone Loss in Rats. J. Periodontol..

