# Supplementary material for: Panax ginseng Fruit Has Anti-Inflammatory Effect and Induces Osteogenic Differentiation by Regulating Nrf2/HO-1 Signaling Pathway in In Vitro and In Vivo Models of Periodontitis
Source: Antioxidants (Basel). 2020 Dec 3;9(12):1221. doi: 10.3390/antiox9121221 (PMC7761716; doi:10.3390/antiox9121221)
Supplement: Supplementary file 1 [file antioxidants-09-01221-s001.pdf]

Supplementary Data

-HPLC-DAD chromatogram-

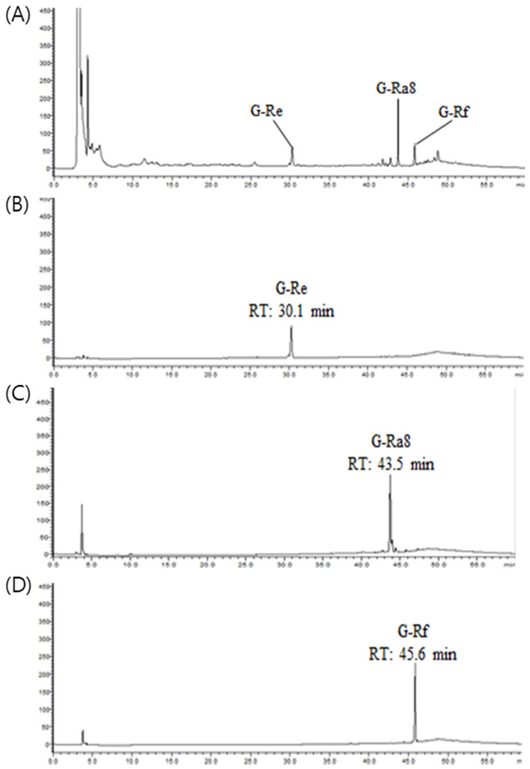

Figure S1. The chromatograms of the *P. ginseng* fruit extract(A) and standard compound solutions G-Re (B), G-Ra8 (C), and G-Rf (D) obtained using HPLC-DAD.
